# Prevalence of Sarcopenia in Connective Tissue Disease Associated Interstitial Lung Diseases: A Single-Centre Study from India

**DOI:** 10.31138/mjr.260423.pof

**Published:** 2024-12-31

**Authors:** M Jeeshitha, Debashis Maikap, Prasanta Padhan, Jayanti Mishra, Pratima Singh

**Affiliations:** 1 Department of Pulmonary Medicine, Kalinga Institute of Medical Sciences, KIIT University, Bhubaneswar, Odisha, India; 2 Department of Clinical Immunology and Rheumatology, Kalinga Institute of Medical Sciences, KIIT University, Bhubaneswar, Odisha, India; 3 Department of Physiology, Kalinga Institute of Medical Sciences, KIIT University, Bhubaneswar, Odisha, India

**Keywords:** sarcopenia, CTD-ILD, 6-minute walk test (6MWT), High Resolution CT (HRCT), thorax

## Abstract

**Background:**

Sarcopenia, a progressive loss of skeletal muscle strength and mass, can lead to decreased quality of life, physical disability, and mortality. Early identification of sarcopenia is crucial in limiting morbidity and mortality in connective tissue disease associated interstitial lung diseases (CTDILD) patients.

**Objective:**

The objectives of this study are to determine the prevalence of sarcopenia in CTD-ILD patients and to correlate the severity of sarcopenia with pulmonary function tests, spirometry, and 6-minute walk test (6MWT).

**Materials and Methods:**

The study involved 32 CTD-ILD patients, documenting their demographic, clinical, and medical history, and conducting various tests, including spirometry, 6MWT, ANA, ENA, MSA profile, and HRCT thorax. Sarcopenia was evaluated using the SARC-F questionnaire, while muscle mass, strength, and physical performance were assessed using the BODYSTAT Quad scan 4000, chair stand test, and gait speed test.

**Results:**

Pre-sarcopenia was the most common condition, followed by sarcopenia and severe sarcopenia. MCTD-ILD and SSc-ILD were the most commonly observed types of CTD-ILD. Patients with pre-sarcopenia had the highest mean FVC, FEV1 (in litres), FVC (%) and FEV1 (%) compared to patients with sarcopenia. The mean distance walked in 6MWT was lowest in patients with severe sarcopenia and highest in patients with sarcopenia, but the difference was not statistically significant.

**Conclusion:**

This study highlights a higher prevalence of sarcopenia in CTD-ILD patients, and its effects on lung function and physical performance. Early identification and intervention for sarcopenia could improve the quality of life and survival in these patients.

## INTRODUCTION

Connective tissue diseases (CTDs) are a spectrum of systemic autoimmune diseases characterised by circulating auto-antibodies and significant autoimmune mediated organ damage. Lungs are affected frequently in connective tissue diseases involving all the compartments of the respiratory system such as respiratory muscles, pleura, conducting airways, lung parenchyma and the small airways, the interstitium or the pulmonary vasculature either separately or in combination with varying clinical presentations. The autoimmune damage to the interstitium of lungs is known as connective tissue disease associated interstitial lung diseases (CTD-ILDs). This may be the initial site of manifestation of the systemic autoimmune disease and contribute significantly to the mortality, morbidity, and economic burden.^1^ In fact, the presence of interstitial lung disease in CTDs has a poor prognosis. The commonly ignored aspect of these diseases is the quality of life experienced by the patient due to musculoskeletal involvement seen in them. Quality of life depends on physical strength which in turn depends on the skeletal muscle mass, muscle strength and its effect on physical function.

The term pre-sarcopenia is defined as loss of muscle mass with preserved muscle strength and sarcopenia is used to describe progressive and generalised loss of skeletal muscle mass and strength with a risk of adverse outcomes such as physical disability, poor quality of life, and death.^2,3^ Because of its effect on mortality and physical disability particularly in patients with connective tissue disorders identification, treatment and prevention of sarcopenia are important to limit its complications. Therefore, we studied the prevalence of sarcopenia and its correlation with determinants of pulmonary functions such as spirometry and 6-minute walk test (6MWT).

## MATERIALS AND METHODS

This cross-sectional observational study was conducted in the departments of Pulmonary Medicine, Clinical Immunology and Rheumatology, and Physiology at Kalinga Institute of Medical Sciences, Bhubaneswar, India from October 2019 to April 2021. The study was approved by the institutional ethics committee (approval number: KIIT/KIMS/IEC/112/2019, Dt. 06/09/2019, approved by KIIT University). As no previous studies were available, we included 32 consecutive cases with radiological and immunological diagnosis of CTD-ILD. Patients with chronic illnesses such as diabetes mellitus, cirrhosis of the liver, chronic kidney disease, congestive cardiac failure, neurological disorders, smokers, myopathies, HIV, Hepatitis B, Hepatitis C, pulmonary tuberculosis, a history of COVID-19 infection, chronic lung diseases except for ILD, and critically ill patients were excluded from the study.

A detailed history and clinical examination using a pre-designed proforma to ensure uniformity of the data was taken from the subjects. Routine blood investigations, including complete blood count, fasting and postprandial blood sugar, kidney function test (KFT), and liver function test (LFT) were performed to rule out any co-morbid illness. HRCT of the thorax was used to identify the presence and pattern of ILD according to radiological classification. All patients underwent sarcopenia assessment, spirometry, and a six-minute walk test.

Sarcopenia assessment was done using the SARC-F questionnaire.^4^ A score greater than or equal to 4 was considered a predictor of sarcopenia and evaluated further. Patients with a score less than 4 were evaluated for pre-sarcopenia. The assessment of patients for sarcopenia was performed using the following methods: Assessment of muscle mass: using multi-frequency BODY STAT Quad scan 4000, which uses the bio-impedance analysis (BIA) principle.^5^

Assessment of muscle strength: Chair stand test.^6^

Assessment of physical performance: Gait speed test.^7^ Bio-impedance analysis (BIA) was performed using the multi-frequency BODY STAT Quad scan 4000 equipment to calculate the details of body analysis and resistance for conductivity at 50 KHz. The resistance value was used to calculate Appendicular Skeletal Mass (ASM) through the prediction equation given by Kim et al.,^8^ for multi-frequency BIA instruments. The ASM was then corrected for height as ASM/ht2.

The Chair stand test involved asking patients to sit in a chair comfortably and then to rise and sit from the chair alternately for five times without the support of hands, with the time taken measured using a stopwatch and noted.

The Gait speed test involved asking patients to walk for four meters at a normal pace, and the time to cover this distance was measured using a stopwatch. The gait speed was calculated by dividing four meters by the time taken to cover this distance.

According to “The Asian Working Group on Sarcopenia”, we defined the cut-offs for sarcopenia as muscle mass <7.0kg/m2 and <5.7kg/m2 for men and women, respectively, using Bio-Impedance Analysis (BIA), and low physical performance as a chair stand test >15 seconds.^9^ Sarcopenia was considered severe if there was a decrease in physical performance along with low muscle mass and strength, defined as gait speed <0.8m/s.^9^

All the patients were subjected to 6MWT and spirometry. Spirometry was performed for all the patients using WinspiroPRO 8.1 software and a MIR minister computer-based spirometer. These values were then classified into different patterns like restrictive, obstructive, and mixed. Restriction in spirometry was graded as per American Thoracic Society (ATS) guidelines into mild, moderate, moderately severe, severe, and very severe.^10^ 6MWT was done by using two arbitrary points at 100-metres from each other by using 2 cones. The procedure was explained to the patient, a digital timer was used, and SpO2 was measured before starting the test, and the patient was asked to walk and SpO2 was measured again after stopping using a pulse oximeter. The 6MWT was performed in room air and patients with resting SpO2 of less than 90% were not taken for the test.

## STATISTICAL ANALYSIS

The study data was collected using a predesigned proforma and then entered into Microsoft Excel 2016 version. IBM SPSS-23.0 software (Chicago, IL, USA) was used for computer-assisted analysis. A p-value of less than 0.05 was considered statistically significant. Categorical data were presented as frequencies and percentages, while continuous data were expressed as mean ± standard deviation (SD).

## RESULTS

The study included 32 cases of CTD-ILD and there were 24 females and 8 males (M: F=1:3). The mean age of the patients was 48.03±14.76 years. In our study, MCTD-ILD (n=13, 40.625%) followed by SSc-ILD (n=11, 34.375%) were the most common subgroups (**Table 1**). Most patients had pre-sarcopenia (n=19; 59.37%) followed by sarcopenia (n=7; 21.9%) and severe sarcopenia (n=6; 18.75%). The mean FVC (litres) and FEV1 (litres) were highest in patients with pre-sarcopenia and lowest in patients with sarcopenia (p=0.11 and 0.07 respectively). Similarly, FVC (%) and FEV1 (%) were highest in patients with pre-sarcopenia (p=0.007 and 0.0049 respectively). Out of 32 patients, 6 could not complete the 6MWT and the mean distance completed in six minutes for patients who were able to complete the test was 347.9 ±64.1 meters. The mean distance walked was lowest in patients with severe sarcopenia (227.4+46.9metres) and highest in patients with sarcopenia (348±142.9 meters) (p=0.1). Parameters like age, BMI, waist circumference, and hip circumference were significantly higher in patients with sarcopenia (p<0.05).

**Table 1.** Types of CTD-ILDs and mean age of presentation.

**Type of CTD-ILD**	**No. of cases (N=32)**	**Percentage (%)**	**Mean age of presentation (age in yrs Mean± SD)**
**MCTD-ILD**	13	40.6	43.3+15.7
**SSc-ILD**	11	34.4	49.8+12
**RA-ILD**	4	12.5	60.75+11.6
**Sjögren’s-ILD**	2	6.3	44.5+3.5
**Overlap CTD-ILD**	1	3.1	22
**Lung-dominant CTD**	2	6.3	57.5+2.5

MCTD-ILD: Mixed connective tissue disease associated interstitial lung disease; SSc-ILD: Systemic sclerosis associated interstitial lung disease; RA-ILD: Rheumatoid arthritis associated interstitial lung disease; ILD-interstitial lung disease; CTD-connective tissue disease.

**Table 2.** Comparison between various parameters in pre-sarcopenia and sarcopenia group.

**Variables (Mean+SD)**	**Pre-sarcopenia (n=19)**	**Sarcopenia (n=13)**	**P value**
**Age in years**	42.7±14	55±13.2	0.012
**BMI (kg/m2)**	22.5±5.2	26±3.4	0.040
**Waist Circumference in cm**	78.6±8.4	88.6±9.7	0.004
**Hip Circumference in cm**	92.5±11	102.2±8.9	0.013
**Disease duration in months**	37.2±30.5	73±85.2	0.168
**Duration of steroid therapy (prednisolone** ≥**7.5mg per day ) in months**	35.6±37.7	37.5±63.7	0.852
**FVC (% predicted)**	67.2±13.6	49.1±18.8	0.003
**FEV1 in % predicted**	64.9±13.34	47.3±17.6	0.002
**FEV1/FVC**	97.5±13.8	98.7±14.3	0.819
**Distance covered in 6MWT (metres)**	318.9±99	292.7±124.3	0.513
**Pointers for SAARC-F questionnaire**	3.26±1.89	4.1±1.3	0.153
**Appendicular skeletal mass (ASM in Kg)**	11.7±2.1	12.2±2.6	0.581
**ASM/HT2 (kg/mt2)**	4.9±0.7	4.9±0.5	0.768
**Chair stand test (seconds)**	12.3±1.9	19.2±2.9	0.0001
**Gait time (metre/second)**	1.17±0.9	0.8±0.2	0.115

* SD: Standard deviation; BMI: body mass index; FVC: forced vital capacity; FEV1: forced expiratory volume in one second; 6MWT: six-minute walk test; ASM: appendicular skeletal mass; HT: height.

**Table 3.** Comparison between various parameters of sarcopenia and severe sarcopenia group.

**Variables (Mean+SD)**	**Sarcopenia (n=7)**	**Severe sarcopenia (n=6)**	**P value**
**Age in years**	52±13	60.3±13.1	0.276
**BMI (kg/m^2^)**	25.5±2.9	26.6±4.1	0.607
**Waist Circumference in cm**	86.1±10.7	91.5±8.3	0.340
**Hip Circumference in cm**	99.9±7.8	105±10	0.320
**Disease duration in months**	53.7±41.2	95.5±119.4	0.402
**Duration of steroid therapy (Prednisolone ≥7.5mg per day) in months**	23.3±23.5	64.8±98.44	0.300
**FVC in % predicted**	44.3±20.1	54.7±17.2	0.343
**FEV1 in % predicted**	41.9±19	53.7±14.7	0.243
**FEV1/FVC**	97.1±16.8	100.5±12.2	0.692
**Distance Covered in 6MWT (metres)**	348.7±50.7	227.4±156.5	0.078
**Pointers for SAARC-F questionnaire**	4.1±1.3	4±1.3	0.847
**Appendicular skeletal mass (ASM in Kg)**	12.1±1.9	12.3±3.4	0.910
**ASM/HT2(kg/mt^2^)**	4.8±0.5	4.9±0.6	0.865
**Chair stand test (seconds)**	19.9±3.2	18.4±2.4	0.361
**Gait time (metre/second)**	1±0.1	0.65±0.14	0.001

* SD: standard deviation; BMI: body mass index; FVC: forced vital capacity; FEV1: forced expiratory volume in one second; 6MWT: six-minute walk test; ASM: appendicular skeletal mass; HT: height.

## DISCUSSION

All patients in our study either had pre-sarcopenia, i.e., decreased muscle mass with preserved muscle strength and physical performance, or sarcopenia. The higher prevalence of pre-sarcopenia in our study could be explained by Rush et al., who stated that South Asians have an overall lower lean muscle mass compared to other European populations due to climatic adaptations and natural variations.

There are no studies on sarcopenia in CTD-ILDs, although a few studies are available on fibrotic ILDs and IPF. In a study conducted by A. Guler et al.^12^ on patients with fibrotic ILDs, and by Maddocks et al.^13^ and Claire M et al.^14^ on IPF patients, the prevalence of pre-sarcopenia and sarcopenia cannot be directly compared with our study. This is because patients with CTD-ILDs exhibit musculoskeletal involvement, leading to a derangement of physical function, a phenomenon not commonly observed in patients with IPF or fibrotic ILDs.

A few studies have investigated the prevalence of sarcopenia in various subgroups of connective tissue diseases without ILD. In our study, the prevalence of sarcopenia in patients with SSc-ILD was higher compared to that reported by Siegert E et al.^15^ in patients with SSc. This difference can be attributed to the concomitant presence of ILD along with SSc in our study. Similarly, in patients with RA-ILD, the prevalence of sarcopenia was higher compared to that reported by Torii M et al.^16^ in rheumatoid arthritis patients, possibly due to the underlying ILD in our study group.

The patients with severe sarcopenia had a prolonged disease duration and steroid usage compared to patients with pre-sarcopenia or sarcopenia. The mean FVC (%) and FEV1 (%) in patients with pre-sarcopenia were higher compared to the values in patients with sarcopenia and severe sarcopenia. However, we saw preserved lung function in patients with severe sarcopenia compared to sarcopenia patients, possibly because of the concomitant use of immunosuppressive and anti-fibrotic drugs along with steroids. Another possible explanation may be limited lung involvement and more musculoskeletal involvement resulting in severe sarcopenia with intact lung function.

In the 6MWT, the mean distance walked was the least in patients with severe sarcopenia compared to people with pre-sarcopenia and sarcopenia. But patients with sarcopenia had a relatively better walking distance compared to the pre-sarcopenia group. The observed variation in our study is likely attributed to three patients in the pre-sarcopenia group unable to complete the 6MWT due to joint pain developed during the test, resulting in a relatively lower mean compared to the sarcopenia group. There are no other comparative studies available for sarcopenia and 6MWT.

In our study population, the mean age showed a significant correlation between pre-sarcopenia and sarcopenia groups (p=0.012), indicating that as age advances, individuals are more likely to develop sarcopenia. This observation aligns with the findings in a study by Goodpaster BH et al.^3^ This significant difference in the age between pre sarcopenia and the sarcopenia groups can act as one of the confounding factors in our study.

In our study, the mean BMI exhibited a significant correlation between the pre-sarcopenia and sarcopenia groups (p=0.04). Patients with sarcopenia had a higher BMI compared to the pre-sarcopenia group, suggesting that a high BMI may be considered one of the risk factors for sarcopenia, contrary to the findings reported by Trajanoska et al.^17^ This discrepancy can be explained by the use of lower limb muscle strength for defining muscle strength, as reported by Curtis M et al.^18^

In our study, the mean waist circumference was found to have a statistically significant difference (p=0.04) between the pre-sarcopenia and sarcopenia groups. Although there are no comparative studies available for this data, Confortin et al.^19^ proposed cut-off values for waist circumference as indicators of sarcopenia (i.e., <88cm for women and <92cm for men). However, these values do not align with our data, and this discrepancy may be attributed to a chance finding due to the small sample size.

In addition, our study revealed a significant association between FVC (%) (p=0.003) and FEV1 (%) (p=0.002) in the pre-sarcopenia and sarcopenia groups, suggesting that patients with sarcopenia had lower lung function compared to the pre-sarcopenia group. This reduced lung function may be linked to hypoxia, leading to a decrease in muscle strength and resulting in sarcopenia. While there are no comparative studies on this specific aspect, Guler et al.^9^ reported a strong association between ILD severity and skeletal muscle mass in fibrotic ILD patients, irrespective of underlying demographics. Despite several limitations, such as a small sample size, being a single-centre study, and a heterogeneous group of patients, sarcopenia was a common finding in CTDILDs. With the continuous advances in available resources, awareness, and medical management, there will be early diagnosis and prolonged survival in patients with CTD-ILD. Therefore, early identification of sarcopenia in these patients is crucial. Measures to prevent sarcopenia are of paramount importance to preserve lung function and ensure an overall good quality of life among these patients.

## CONCLUSION

The high prevalence of sarcopenia, coupled with decreased lung function and impaired physical performance, has a significant impact on the overall quality of life, morbidity, and mortality in patients with CTD-ILD.
